# Vitamin D-Related Risk Factors for Maternal Morbidity during Pregnancy: A Systematic Review

**DOI:** 10.3390/nu14153166

**Published:** 2022-07-31

**Authors:** Maria Morales Suárez-Varela, Nazlı Uçar, Isabel Peraita-Costa, María Flores Huertas, Jose Miguel Soriano, Agustin Llopis-Morales, William B. Grant

**Affiliations:** 1 Area of Preventive Medicine and Public Health, Department of Preventive Medicine and Public Health, Food Sciences, Toxicology and Legal Medicine, School of Pharmacy, University de Valencia, Avenida Vicent Andres Estelles s/n, 46100 Valencia, Spain; maria.m.morales@uv.es (M.M.S.-V.); unaz@alumni.uv.es (N.U.); ipecos@alumni.uv.es (I.P.-C.); marcia.f.huertas@uv.es (M.F.H.); allomo@alumni.uv.es (A.L.-M.); 2 Biomedical Research Center Network on Epidemiology and Public Health (CIBERESP), Institute of Health Carlos III, Avenida Monforte de Lemos, 3-5, Pabellón 11, Planta 0, 28029 Madrid, Spain; 3 Unit of Nutrition and Bromatology, Department of Preventive Medicine and Public Health, Food Sciences, Toxicology and Forensic Medicine, Universitat de València, Avda. Vicente Andrés Estellés s/n, Burjassot, 46100 Valencia, Spain; jose.soriano@uv.es; 4 Sunlight, Nutrition, and Health Research Center, P.O. Box 641603, San Francisco, CA 94164-1603, USA

**Keywords:** gestational diabetes, hypertension, maternal morbidity, preeclampsia, pregnancy, supplementation, vitamin D, 25-hydroxyvitamin D

## Abstract

Vitamin D has well-defined classical functions related to metabolism and bone health but also has non-classical effects that may influence pregnancy. Maternal morbidity remains a significant health care concern worldwide, despite efforts to improve maternal health. Nutritional deficiencies of vitamin D during pregnancy are related to adverse pregnancy outcomes, but the evidence base is difficult to navigate. The primary purpose of this review is to map the evidence on the effects of deficiencies of vitamin D on pregnancy outcome and the dosage used in such studies. A systematic search was performed for studies on vitamin D status during pregnancy and maternal outcomes. A total of 50 studies came from PubMed, 15 studies came from Cochrane, and 150 studies came from Embase, for a total of 215 articles. After screening, 34 were identified as candidate studies for inclusion. Finally, 28 articles met the inclusion criteria, which originated from 15 countries. The studies included 14 original research studies and 13 review studies conducted between 2012 and 2021. This review was finally limited to the 14 original studies. This systematic review was conducted according to the Preferred Reporting Items for Systematic Review and Meta-Analysis (PRISMA) guidelines, and the quality and strength of the evidence was evaluated using the Navigation Guide Systematic Review Methodology (SING). We found evidence that supports the idea that supplementary vitamin D for pregnant women is important for reducing the risk of gestational diabetes, hypertension, preeclampsia, early labor, and other complications. The data retrieved from this review are consistent with the hypothesis that adequate vitamin D levels might contribute to a healthy pregnancy.

## 1. Introduction

There is evidence of early interest in the relationship between vitamin D status and maternal health outcomes [1].

Vitamin D (D_2_ or ergocalciferol, D_3_ or cholecalciferol, or both) is a fat-soluble lipophilic prohormone proven to have many metabolic and biological functions. This vitamin is mainly synthetized in the skin as cholecalciferol through the action of ultraviolet light (vitamin D3), but it is also obtained from diet sources and food supplements such as ergocalciferol (vitamin D2) [2] and food materials such as fish oil, fish flesh, dietary supplements, eggs, butter, fortified foods, liver, and mushrooms. Vitamin D deficiency (serum 25-hydroxyvitamin D [25(OH)D] < 20 ng/mL) [3,4,5] is a major public health concern that is widespread among the general population and highly prevalent in pregnant women; it is found in 60% of them [6,7,8,9]. Maintaining serum concentrations between 30 and 50 ng/mL is recommended to achieve the health benefits of vitamin D [10,11,12,13].

Globally, it has been estimated that a billion people may be affected by vitamin D deficiency or insufficiency [14]. Studies in Ethiopia and India have also found that more than 80% and 60% of pregnant women suffered from vitamin D deficiency, using a cutoff of <50 nmol/L vitamin D, indicating the need for more research on the potential outcome and benefits of supplementation in developing countries [15,16].

Severe maternal morbidity during pregnancy is identified and reported worldwide. Its rising rates remain a large healthcare concern [17]. In 2005, worldwide, there were around 535,900 maternal deaths reported, which translates to a mortality ratio of about 402 maternal deaths per 100,000 live births [18]. The majority of these maternal deaths occurred in sub-Saharan Africa, with 270,500 deaths, and Asia, with 240,600 deaths [18]. Just five countries—India (117,100), Nigeria (58,800), the Democratic Republic of Congo (32,300), Afghanistan (26,000), and Ethiopia (22,200)—accounted for almost half (48%) of all maternal deaths [18].

Maternal morbidity is an unintended outcome of labor and delivery that results in significant short- or long-term consequences to woman’s health [19]. Severe maternal morbidity (SMM) affects around an estimated 50,000 women per year in the United States—0.5–1.3% of pregnancies [19,20]. However, determining the true rates of SMM in the United States and worldwide is difficult because of the lack of standard definitions of such cases as well as the difficulty in identifying cases [21].

During pregnancy, there are significant alterations in phosphate and calcium metabolism owing to calcium accumulating in the fetal skeleton, and the fetus relies exclusively on the maternal supply of vitamin D, which it receives across the placenta, as it is not capable of synthesizing vitamin D on its own for adequate bone mineral formation [22,23]. A low level of vitamin D during the pregnancy and special attention during the early stage of pregnancy produce less bone mineral content in the fetal skeleton. Calcitriol cord blood concentrations tend to be lower than those found in maternal serum [2,3,4,5,6,7,8,9,10,11,12,13] due to the fact that calcitriol cannot easily cross the placental barrier [24,25], and parathyroid hormone concentrations are low in the fetus [26]. The high levels of phosphorus and calcium concentrations found in serum also contribute to lower fetal calcitriol concentrations because these factors suppress the expression of renal 25OHD-1-α-hydroxylase (CYP27B1) in the fetus [27].

The recommended daily allowance (RDA) of vitamin D for women in the United States aged 19–50 years, including during pregnancy, is established at 600 IU per day [27]. This recommendation was based on the amount of intake necessary to sustain blood levels of vitamin D above 50 nmol/L for a population with minimal sunlight exposure and was developed solely based on outcomes related to bone health [27]. According to the US Institute of Medicine, it is considered that 1000–1600 IU (25–40 g/day) of supplemental vitamin D is necessary during pregnancy to obtain the highest level of vitamin D3 during this period [28]. This recommendation was contentious, as many researchers have argued that insufficiency should be defined at thresholds of 75 nmol/L or even higher, which would require a much higher intake to reach [29,30]. Nevertheless, some studies [31,32,33] established that the safe and maximal production of vitamin D (at least 32 ng/mL) is achieved with a supplementation of 4000 IU/day until delivery.

Vitamin D can also be referred to as 25-hidroxyvitamin D or calcidiol, and it is transformed into its active form 1,25-dihydroxyvitamin D by CYP27B1 [33]. This enzyme is mainly located in the kidney but is also significantly expressed in the placenta. Pregnancy represents a special physiological situation due to the important role played by the placenta in the metabolism of this vitamin [34]. The placenta is thought to be the major site of vitamin D metabolism in pregnancy. The 1a-hydroxylase, the 24-hydroxylase, the 25-hydroxylase (CYP2R1), the vitamin D binding protein (VDB), and the vitamin D receptor (VDR) have all been detected either in trophoblast cultures or in freshly obtained placental tissue [35,36,37,38]. Undoubtedly, the placenta can metabolize vitamin D, providing active 1,25-(OH)2 vitamin D in vitro. However, it is unclear to what extent placental vitamin D metabolism contributes to maternal vitamin D status in pregnancy.

Numerous functions have been attributed to vitamin D due to the pleiotropic properties of the vitamin D receptor (VDR) [39]. Increasing scientific evidence points to the role of vitamin D in maternal mortality and morbidity, in addition to its implication in several pathologies. Allergic and autoimmune diseases and even cancer implications have also been postulated [40]. The vitamin D deficiency during pregnancy cause maternal and fetal side effects [41], such as increases the risk of preeclampsia, glucose intolerance, gestational diabetes, preterm birth and hypocalcemia crisis in the mother. As poor skeletal development, dysfunction in both the mother and newborn and increase the risk birth of a small child for gestational age (SGA) [42]. Also in the fetus it is related to an inadequate immune system, wheezing and eczema, and respiratory infections in infants [43,44].

An area of study that has garnered significant attention is the role of vitamin D and its effect on pregnancy. There is a lack of evidence from systematic reviews and meta- analyses to evaluate the association between vitamin D during pregnancy and maternal morbidity. Given the high prevalence of low vitamin D level status during pregnancy and the public health importance of clarifying the role of vitamin D during pregnancy in offspring health, a better understanding of the nonclassical functions of vitamin D in preventing adverse health outcomes in high-risk populations is needed. The aim of the present review is to summarize the primary outcome in order to identify a cut-off value for a serum vitamin D concentration that increases the risk of maternal morbidity during pregnancy and to determine the possibility of supplementation to avoid it.

## 2. Materials and Methods

This systematic review was conducted according to the Preferred Reporting Items for Systematic Reviews and Meta-Analyses (PRISMA) guidelines [45,46]. The quality and strength of the evidence was evaluated using the Navigation Guide Systematic Review Methodology (SING) [47,48,49]. Systematic review registration PROSPERO (CDR42022343174). 

### 2.1. Question PECO

The PECO question (P: population; E: exposure; C: comparison; O: outcome) of the study was “Is there more morbidity in pregnant women with low levels of vitamin D compared to those with adequate levels of vitamin D?”, in which P is pregnancy women; E is a low intake/level of vitamin D; C is an adequate intake/level of vitamin D; and O is pregnancy morbidity. 

### 2.2. Literature Search

**Figure 1:**
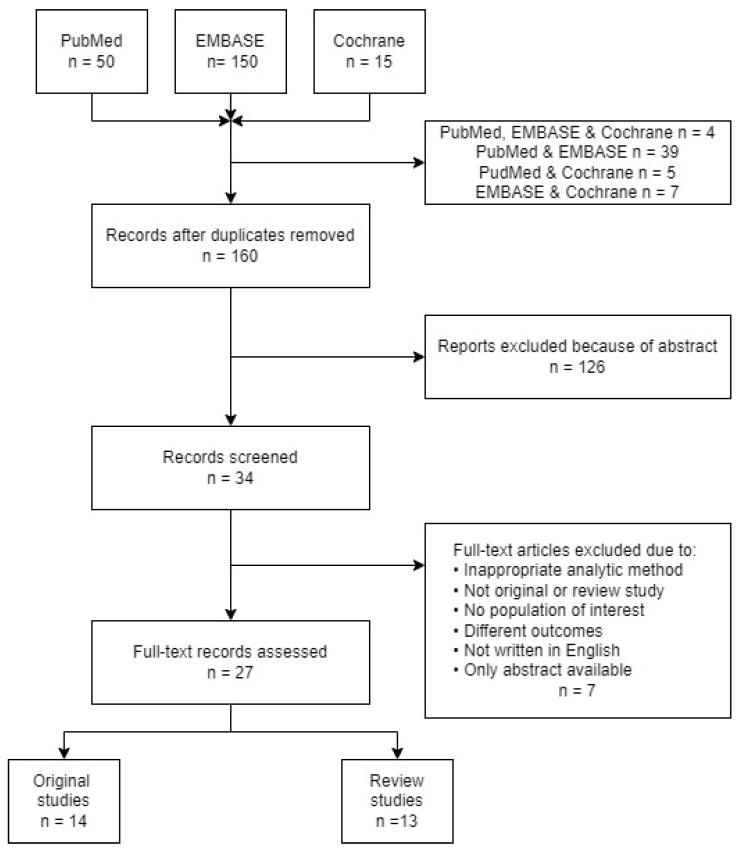
Search strategy: PRISMA flowchart.

The goal of the search strategy was to identify studies that reported the associations between serum vitamin D concentrations or the intake of vitamin D from supplementation or diet during pregnancy and its maternal morbidity affects. First, we performed a literature search to identify publications eligible for inclusion in the PubMed and Embase databases. The keywords included “pregnancy” OR “gestation” AND “vitamin D” AND “morbidity.” The search was limited to human subjects and English and Spanish language articles published between 2010 and May 2022. A total of 50 studies were recovered from PubMed, 15 were recovered from Cochrane, and 150 were recovered from Embase, for a total of 215. In the first phase, duplicates were removed, and the reference lists of relevant publications were searched for fresh research that fulfilled the inclusion requirements. Following the first literature search, the reviewers examined the titles and abstracts to locate those that fulfilled the selection criteria. These articles were assessed for eligibility, with the first screening of the articles based on the information available in the abstract and results sections of each study. The initial screening identified 34 candidate studies, of which 28 met the inclusion and exclusion criteria. The PRISMA flowchart (Figure 1) shows the number of articles at each stage of the screening process. 

### 2.3. Study Inclusion/Exclusion Criteria and Data Extraction

The types of studies included in this review meet the following criteria: controlled trials, both randomized and nonrandomized; prospective cohorts; case-control studies; and systematic reviews looking at the effects of vitamin D on maternal morbidity. All studies were longitudinal in nature and focused on how vitamin D levels in pregnancy were related to maternal morbidity. Specific inclusion/exclusion criteria were developed for the selection of studies to be included in this work, and only published works that met all the criteria were included for review. The selection criteria were the following:

1. Original research article or review (abstracts, case reports, ecological studies, and comments were excluded)
2. Available in English and Spanish
3. Published between 2010 and May 2022
4. Study carried out on humans
5. Exposure of interest is vitamin D status or supplementation during pregnancy
6. Data on vitamin D or metabolite concentration in maternal blood during pregnancy available
7. Main outcomes of interest are the incidence of maternal morbidity.

After a thorough assessment by all the authors of the candidate studies, 26 were included in this review.

### 2.4. Data Extraction

The data for the present review were retrieved from the previous research articles published earlier. The following data were extracted for the present study: (i) Study characteristics: authors, location and year, type of study, and source of data collection; (ii) sample size; (iii) primary outcome; (iv) findings (maternal morbidity & vitamin D level) (Table 1). The relevant data of the reviews were also summarized in a second table, including: (i) factors analyzed; (ii) gestational week when sample was collected; (iii) vitamin D cutoff (blood sample nmol/L); (iv) vitamin D collected (serum or supplementation); (v) average maternal age (Table 2).

### 2.5. Study Quaity Assessment

The quality of the studies is assessed using the following tools: The Eight Star Newcastle–Ottawa Scale (NOS) for observational studies (cohorts and case-controls) [47,48] was used to evaluate the methodological quality—specifically, the risk of bias—of the original studies. Assessment with the Newcastle–Ottawa Scale produces a score ranging from 0 to 9, with the overall score based on three sub-scores based on the subject selection (0–4), the comparability of the subject (0–2), and the clinical outcome (0–3). The study assessment was carried out independently by two individuals (NU and IPC), and discrepancies were brought to a third individual (MMSV) if a compromise could not be reached among the two original individuals after discussion.

Further assessment of the quality of the included studies was carried out using the Scottish Intercollegiate Guidelines Network (SIGN) [49]. Using the SIGN ensures that the validity—including key factors such as bias and confounding—of a study is robustly assessed. The SIGN system in based on the principles of evidence-based medicine, an approach that ensures the use of the most up-to-date, reliable, and scientifically solid evidence available in making decisions about a particular situation being studied [64].

The SIGN system establishes levels of evidence and recommendations to describe a given study and its results. The levels of evidence are based on the study design and the methodological quality of individual studies and are scored from best to worst using the numbers 1, 2, 3, and 4. These scores are further ranked using the ++, +, and—signs. The grades of recommendation, rated from best to worst as A, B, C, and D, are based on the strength of the evidence on which the recommendation is based, and they do not reflect the clinical importance of the recommendation.

## 3. Results

### 3.1. Study Characteristics

Our search approach yielded up 215 studies identified through database searching; a total of 14 original research studies and 13 review studies remained. After consideration, it was decided to include only the 14 original studies in this review.

Considering the SIGN and NOS scores, the 14 original studies could be regarded as good (high) quality. The important methodological features and the general characteristics of all the review studies are summarized in Table 1. The chosen studies were analyzed according to the design, location and year, source of data, sample size, factor, vitamin D level assessment, and major findings. Meanwhile, the vitamin D analysis details and vitamin D cutoff values of the included articles are listed in Table 2.

The studies were published between 2012 and 2021. The original research studies used data from India [57,58,61], Denmark [59], the United States [54,56], Germany [55], Nigeria [53], the Northern Hemisphere [51], Canada [52], Brazil [50], Egypt [60], Sweden [62], and Mexico [63]. The review research studies included data from Brazil, India, the United States, Puerto Rico, Spain, Iran, and Australia [65,66,67,68,69,70,71,72,73,74,75,76,77].

All but six observational studies of vitamin D were conducted in high-income country settings, and most populations had either a presumed risk or a high prevalence of deficiency at baseline (Table 1). The dosing approaches and assay methods in the trials varied: one trial contained multiple intervention arms testing the daily dietary intake of Vitamin D, vitamin D supplementation, and the frequency of UV exposure in the first trimester, in the second trimester, and at the time of delivery. One recent trial tested daily 4400 vs. 400 IU D3. In other studies, the relationship between disease risks was evaluated by measuring serum vitamin D levels with different assay methods (Table 2). This trial [65] showed that a significant effect of sufficient vitamin D status (25OHD ≥ 30 ng/mL) was observed in both early and late pregnancy compared with insufficient levels (25OHD < 30 ng/mL) (OR, 0.28; 95% CI, 0.10–0.96).

Vitamin D supplementation appeared to improve maternal vitamin D levels in the two trials for which data were available [65]. In addition, the results of trials by Christine Rohr Thomsen indicate a seasonal variation effect of the risk of gestational hypertension (*p* = 0.01), PE (*p* = 0.001), and early-onset PE (*p* = 0.014) [51,59]. Women with an estimated date of conception in June had the highest risk of preeclampsia, while women with an estimated date of conception in August had the highest risk of gestational hypertension.

Observational studies of vitamin D status during pregnancy and the risk of pre-eclampsia have not shown consistent associations. Vitamin D levels were lower (*p* < 0.01) in women with PE [50,51,52,57,58,60,61]. The investigators of a study from the USA [54] observed that vitamin D supplementation initiated in weeks 10–18 of pregnancy did not reduce preeclampsia incidence in the intention-to-treat paradigm. However, vitamin D levels of 30 ng/mL or higher at trial entry and in late pregnancy were associated with a lower risk of preeclampsia (8.08% vs. 8.33%, respectively; relative risk: 0.97; 95% CI, 0.61–1.53). A nested case control study from North Carolina reported that women with vitamin D levels <50 nmol/L had a nearly fourfold greater risk of severe preeclampsia compared with those with levels ≥ 75 nmol/L [78]. In contrast, a nested case-control study in Massachusetts found no statistically significant differences in the risk of pre-eclampsia for women with vitamin D levels < 37.5 nmol/L (AOR 1.35 [0.40, 4.50]) [71]. Another prospective cohort study of pregnancies at a high risk for pre-eclampsia in Canada found no effect of vitamin D during early pregnancy on pre-eclampsia risk [72].

A group of studies relate the vitamin D status with the alteration of different metabolic pathways such as carbon and peptide metabolism. The imbalance of long-chain polyunsaturated fatty acid metabolites produced by a vitamin D deficiency contributes to inflammation and endothelial dysfunction [61]. This deficiency also contributes to a low antimicrobial peptide metabolism [63], resulting in several urinary infections.

### 3.2. Original Research Studies

Nandi and colleagues [58] published a cross-sectional study in 2019. The study included 119 pregnant women (69 normotensive controls [NC] and 50 women with PE). The women with PE had lower maternal and cord serum vitamin D levels (*p* < 0.01 for both) than the NC women. A total of 94% of women in the PE group and 76% in the NC group were deficient in maternal vitamin D levels, while for cord vitamin D levels, 98% of women with PE and 85.2% of NC women were deficient. In 2020, this group reported [61] how the imbalance in the long-chain polyunsaturated fatty acid (LCPUFA) metabolites derived from vitamin D deficiency contributes to placental inflammation and endothelial dysfunction in PE.

Rohr Thomsen and colleagues [59] published a cohort study based on data from the Aarhus Birth Cohort (ABC). Of the 50,665 women included, 4285 (8.5%) were diagnosed with a hypertensive disorder of pregnancy, 1999 (3.9%) were diagnosed with PE, and 2386 were diagnosed (4.7%) with gestational hypertension (GH). The hypertensive disorders of pregnancy, including GH, PE, and early-onset PE, increased the risk for women conceiving during spring and early summer, peaking in midsummer, and later decreasing steadily during late summer and fall to reach the nadir by winter. Seasonal variation was found for GH (*p* = 0.01), PE (*p* = 0.001) and early-onset PE (*p* = 0.01). In another prospective comparative study [68], a significant negative correlation was observed between vitamin D and systolic and diastolic blood pressure in the PE group (*p* < 0.05), whereas no significant correlation was observed between vitamin D and systolic/diastolic blood pressure in the control group. The mean vitamin D level was significantly lower in the PE group than that in the control group (9 ± 5 and 14 ± 8 ng/mL, respectively), with a statistically significant *p* < 0.05. A vitamin D level < 5 ng/mL was associated with a 14.58-fold (95% CI; 12.16–17.55) increase in the odds ratio of PE, whereas a vitamin D level of 5–10 ng/mL was associated with an 11.42-fold (95% CI; 8.26–13.6) increase in the odds ratio of PE.

In 2017, Accortt and colleagues [56] found an association between a higher postpartum allostatic load and an index of multisystem physiological wear and tear, operationalizing emergent chronic disease risk and predicting morbidity and vitamin D. Adding vitamin D deficiency to the allostatic load index produced a stronger association with adverse outcome. Brodowski and colleagues [55] assessed the effect of vitamin D supplementation (4400 vs. 400 IU/day) initiated early in pregnancy (10–18 weeks) on the development of PE. When started at weeks 10–18 of pregnancy, vitamin D supplementation did not reduce the incidence of PE. However, vitamin D levels of ≥30 ng/mL at trial entry and in late pregnancy were associated with a lower risk of PE.

Lawal and colleagues [53] showed that no relationship exists between vitamin D deficiency and GDM. That case-control study had 200 pregnant women; the proportion of cases (*n* = 100) and controls (*n* = 100) with vitamin D insufficiency was 62% and 54%, respectively. Lechtermann and colleagues [51] indicated that patients with PE had lower serum levels of vitamin D in response to seasonal changes.

In 2020, Schoenmakers and colleagues [62] found a correlation between a relatively high concentration of 1,2(OH)2D and hypercalcemia in pregnant women during the third trimester. The retrospective and explorative study investigated the prevalence of hypercalcemia in a cohort of 2121 women—1827 screened for hypercalcemia in T3. The prevalence was 1.7% higher than that in the general population.

Olmos-Ortiz and colleagues suggest [64] cardiovascular risk and perinatal infections due to vitamin D3 (calcitriol) deficiency, especially in male-carrying pregnancies due to the lower calcitriol-activating enzyme. The placental calcitriol was significantly elevated in women with urinary tract infections, and it was negatively correlated with blood pressure. Regarding newborns’ sex, the calcitriol-activating enzyme showed a higher expression in female-carrying mothers.

The level of evidence is relatively high—2++ or 2+, according to SIGN, which belong to a great level of recommendation: B. The systematic review about the importance of the maintenance of a good level of vitamin D could be used as a recommendation guide in the studied population: pregnant women.

## 4. Discussion

Overall, this systematic review suggests that maternal low levels of vitamin D during pregnancy lead to a greater risk of gestational diabetes, preeclampsia, early labor, and other complications. However, due to the variability in numerous elements of the study design (e.g., vitamin D assessment methods, pregnant mobility assessment methods, and the timing of the data collection), it remains a challenge to synthesize the findings. This data suggest that low maternal vitamin D appears to have a negative impact or detrimental impact on the health status of pregnant women, which is an important conclusion that prevents many women from getting adequate nutrition with the adequate support of vitamin D, and it is not possible to use supplementation during the pregnancy period.

Recently, vitamin D has been recognized as interacting with a nuclear receptor in various organs [71,72,73,74,75,76]. Vitamin D deficiency is associated with increased risks of morbidity and mortality in cardiovascular, malignant, and autoimmune diseases [72,77,78]. In recent years, the interest in the consequences of maternal vitamin D deficiency and its effect on pregnancy has increased. Vitamin D insufficiency is considered common in pregnant women, and deficiencies have been linked to adverse pregnancy outcomes [78,79,80].

Considering whether prenatal vitamin D deficiency is associated with maternal morbidity seems reasonable. The findings from several studies suggest an increasing prevalence of vitamin D deficiency in pregnancy and its associated adverse outcomes [81,82,83,84,85]. To further understand the role of vitamin D in pregnancy and the seemingly associated adverse outcomes, interventional and observational studies are needed.

Furthermore, a current systematic review described the overall mean prevalence rates of vitamin D deficiency in pregnant women and newborns as 54% and 75%, respectively [86]. In postpartum periods, the prevalence of vitamin D deficiency in women is also high: 63% [86,87]. Although evidence points to the high prevalence of deficiency, there exist strategies to raise maternal vitamin D concentrations, including supplementation, advice for sun exposure (15–20% of the body surface area), and the intake of vitamin D–fortified foods. The vitamin D status during pregnancy varies around the world as a function of maternal sunlight exposure, the degree of skin pigmentation, latitude, lifestyle, BMI, and the intake of vitamin D supplements. People with darker skin pigmentation and limited sunlight exposure are at the greatest risk for deficiency [88].

Supplement intake can also play an important role in improving vitamin D status among pregnant women. Taking vitamin D-enriched food and supplements can be advised in order to maintain optimum serum levels during pregnancy. The recommendations for vitamin D intake during pregnancy range from 200 to 4000 IU/day worldwide. The current WHO guideline recommends 200 IU/day of vitamin D supplement intake among pregnant women with vitamin D deficiency in order to reduce the risk of PE, a low birth weight, and a preterm birth [89]. The American Pregnancy Association recommends 100 μg/day of vitamin D intake, a considerably larger amount of vitamin D than the recommended intake of 10 μg/day for women [90]. In China, a daily intake of 600 IU is suggested during pregnancy [91]. In the United Kingdom, it is advised to have a maternal vitamin D intake of 400 IU/day. The United Kingdom Health Department provides free vitamin D supplementation to pregnant women and newborn children [92]. Switzerland follows the Institute of Medicine-recommended nutrient intake: 1500–2000 IU/day for women at risk of vitamin D deficiency and 600 IU for women without such risk [93]. In Canada, pregnant women are suggested to take 400–600 IU/day [94]. In Turkey, free supplementation of vitamin D (1200 IU/day) is provided to all women from early pregnancy to 6 months after delivery [95]. A similar approach to vitamin D supplementation (400 IU/day) is followed in New Zealand for pregnant women identified as being at risk of vitamin D deficiency [96]. Meanwhile, for women not at risk, the ministry of health of New Zealand recommends 200 IU/day [97,98,99].

After many years of study, researchers at the Medical University of South Carolina College of Medicine suggested 4000 IU/day of vitamin D for pregnant women. The findings suggest that, starting at 12–16 weeks of gestation, vitamin D supplementation at a rate of 4000 IU/day is most effective in achieving vitamin D sufficiency in order to attain an optimal nutritional and hormonal vitamin D status throughout pregnancy [88]. A treatment (<37 weeks) goal > 40 ng/mL was associated with a reduction in preterm birth risk [31].

Further, no trials or observational studies specifically regarding vitamin D supplementation/intake and maternal morbidity during pregnancy were identified. Nevertheless, vitamin D requirements are higher among pregnant women, and maintaining optimum serum levels of vitamin D during maternity and for fetus growth is important. Adequate levels of vitamin D seem to be a determinant at the time of implantation and placentation for the development of preeclampsia. There is not a consensus regarding the vitamin D blood concentration value that predisposes women to maternal morbidity; hence, is not easy to recommend a specific supplementation treatment. The present systematic review lacks the experimental data needed to establish a general cutoff value of vitamin D in order to settle how important it could be to improving the maternal diet with vitamin D supplements. Further exploration of vitamin D’s role in pregnancy and its potential role in maternal morbidity would be worthwhile, including maternal age and sexual dimorphism.

## 5. Strengths and Limitations of This Review

This study has limitations. First, there were limited data on maternal vitamin D supplementation during pregnancy regarding long-term outcomes. Second, the studies included here show significant methodological differences, which problematizes the obtention of a consensus on the evidence currently available on the relationship between vitamin D and maternal morbidity during pregnancy. In addition, we may not have been able to access all publications on the relationship between vitamin D and maternal morbidity during pregnancy because the area of analysis is limited to studies that are published in English and Spanish and that are available through the PubMed, Cochrane, and Embase databases.

## 6. Conclusions

Despite the inherent limitations discussed above that limit the ability to draw conclusions across studies, some important findings were noted. Collectively, the studies suggest that appropriate levels of vitamin D during pregnancy are associated with less mobility during pregnancy. Pregnant women should be counselled to maintain an adequate intake of vitamin D, with suitable nutritional support to adequately control their levels. In this systematic review of the literature, we found evidence relating vitamin D to maternal morbidity-related outcomes. However, well-designed, randomized vitamin D supplementation trials in pregnant women carried out to determine the optimal vitamin D status and dosing and evaluate the potential effectiveness of supplementation with respect to the risk of maternal morbidity are still greatly needed.

## Figures and Tables

**Table 1:** Original studies that show vitamin D-related risk factors for maternal morbidity during pregnancy.

Author	Location, Year(s)	Study Type	Data Source	Sample Size	Primary Outcome	Findings	SING&		NOS
							LE	GR	
Rezende et al., 2012 [50]	Brazil	Case-control; observational	IRB at the Faculty of Medicine of Ribeirao Preto, University of São Paulo	*n* = 529: *n* = 154 (GH) *n* = 162 (PE) *n* = 213 (healthy)	PE and GH	Similar genotype distributions were found for the 3 VDR polymorphisms in both the PE and GH groups compared with the HP group (all *p* > 0.05). VDR haplotype frequency distribution was similar in both the PE and GH groups compared with the HP group (all *p* > 0.05).	2^++^	B	8
Lechtermann et al., 2014 [51]	Northern Hemisphere, 2005–2008	Cohort; observational	Department of Gynecology and Obstetrics, UK-Essen, University of Duisburg-Essen, Germany	*n* = 63: *n* = 20 (PE) *n* = 43 (healthy)	PE	In patients with PE, vitamin D levels were lower but differed significantly from the controls only in the summer (18.21 ± 17.1 vs. 49.2 ± 29.2 ng/mL; *p* < 0.001), whereas 1,25-(OH)_2_ vitamin D levels were significantly lower only in the winter (291 ± 217 vs. 612.3 ± 455 pmol/mL; *p* < 0.05). A two-factorial ANOVA produced a statistically significant model (*p* < 0.0001) with an effect of season (*p* < 0.01) and PE (*p* = 0.01) on maternal vitamin D levels, as well as a significant interaction between the two variables (*p* = 0.02).	2^++^	B	8
Achkar et al., 2015 [52]	Canada, 2014	Nested case-control	Canadian cohort studies of pregnant women, Quebec City, Nova Scotia, and Halifax, 2002–2010	*n* = 169 (PE) *n* = 1975 (control)	PE	Women who developed PE had a significantly lower vitamin D concentration (47.2 ± 17.7 vs. 52.3 ± 17.2 nmol/L; *p* < 0.0001). Women with vitamin D <30 nmol/L, compared with those with at least 50 nmol/L, had a greater risk of developing PE (adjusted OR = 2.23; 95% CI, 1.29–3.83) after adjustment for pre-pregnancy BMI, maternal age, smoking, parity, season and year of blood collection, gestational week at blood collection, and cohort site. An exploratory analysis with cubic splines showed a dose–response relationship between maternal vitamin D and the risk of PE, up to levels ~50 nmol/L, where the association appears to plateau.	2^++^	B	8
Lawal et al., 2016 [53]	Nigeria, 2014	Case-control; observational	Department of Chemical Pathology of the tertiary health care facility	*n* = 100 (GDM) *n* = 100 (control)	GDM	Overall mean values of plasma 25-hydroxycholecalciferol were 28.77 ± 12.42 ng/mL. Overall, 58% of subjects had plasma 25-hydroxycholecalciferol levels < 30 ng/mL. The proportion of cases with vitamin D insufficiency was 62% (54% for controls). The OR for GDM was 1.39 (95% CI, 0.79–2.44) and *p* = 0.3159.	2^++^	B	8
Mirzakhani et al., 2016 [54]	USA, 2009–2011	Randomized, double-blind, placebo-controlled clinical trial; experimental	Boston University Medical Center; Washington University in St. Louis, Missouri; and Kaiser Permanente Southern California Region in San Diego	*n* = 440 (4400 IU) *n* = 436 (placebo 400 IU)	PE	No significant difference was found between the treatment or control groups in terms of incidence of PE (8.08% vs. 8.33%, respectively; relative risk: 0.97; 95% CI, 0.61–1.53). In a cohort analysis and after adjustment for confounders, a significant effect of sufficient vitamin D status (≥30 ng/mL was observed in both early and late pregnancy compared with insufficient levels (adjusted OR, 0.28; 95% CI, 0.10–0.96). The differential expression of 348 vitamin D-associated genes (158 upregulated) was found in the peripheral blood of women who developed PE (FDR <0.05 in the Vitamin D Antenatal Asthma Reduction Trial [VDAART]; *p* < 0.05 in a replication cohort).	2^++^	B	8
Brodowski et al., 2017 [55]	Germany	Cohort; observational	Hannover Medical Center (Germany)	*n* = 12 (PE) *n* = 13 (NC)	PE	Vitamin D_3_ improved HUVEC function in neither group. No effect of vitamin D_3_ on VEGF expression was found.	2^++^	B	8
Accortt et al., 2017 [56]	USA, 2004–2016	Nested cohort; observational	Community Child Health Network	*n* = 164 (cohort)	PE and GDM	Serum vitamin D was significantly inversely correlated with the AL index (Spearman’s *r* = −0.247; *p* = 0.002).	2^+^	B	8
Singla et al., 2019 [57]	India, 2017–2018	Prospective comparative; observational	Department of Obstetrics and Gynaecology, Adesh Institute of Medical Sciences and Research, Bathinda, Punjab	*n* = 60: *n* = 30 (PE) *n* = 30 (NC)	PE	Vitamin D deficiency was found in all participants, but the mean vitamin D level was significantly lower in the PE group (8.7 ± 5.32 vs. 14.2 ± 7.88 ng/mL, *p* < 0.05).	2^++^	B	8
Nandi et al., 2020 [58]	India	Cross-sectional; observational	Department of Obstetrics and Gynecology, Bharati Medical College and Hospital, Pune	*n* = 50 (PE) *n* = 69 (NC)	PE	Vitamin D levels were lower (*p* < 0.01 for both) in women with PE. PUFA levels were lower (*p* < 0.05), whereas SFA and total MUFA were higher (*p* < 0.05 for both) in women with PE. Cord erythrocyte PUFA levels were higher (*p* < 0.01) in PE women. Vitamin D levels were negatively associated with maternal systolic and diastolic blood pressure (*p* < 0.01 for both). Vitamin D levels were positively associated with PUFA (*p* < 0.01) and negatively associated with SFA (*p* < 0.05), MUFA (*p* < 0.01).	2^++^	B	8
Rohr Thomsen et al., 2020 [59]	Denmark, 1989–2010	Cohort; observational	Aarhus Birth Cohort at the Department of Gynecology and Obstetrics, Aarhus University Hospital	*n* = 50,665 (cohort)	GH and PE	Seasonal variation was found for GH (*p* = 0.01), PE (*p* = 0.001), and early-onset PE (*p* = 0.014). Increased risk was observed when conceiving during spring and early summer, peaking in midsummer, and decreasing steadily during late summer and fall to reach the nadir by winter.	2^++^	B	8
Osman et al., 2020 [60]	Egypt, 2019	Case-control; observational	—	*n* = 200 (PE) *n* = 100 (eclampsia) *n* = 200 (NC)	Eclampsia and PE	Mean vitamin D level was lower in the PE group (14.8 ± 5.4 ng/mL) and the eclampsia group (10.5 ± 1.6 ng/mL) than in the pregnant controls (19.5 ± 6.5 ng/mL) (*p* = 0.002). The difference was significant only between the eclampsia group and the pregnant controls (*p* = 0.02). All eclampsia cases had vitamin D insufficiency, compared with 17.5% of the PE group and 39.5% of the controls. Deficiency of vitamin D (<12 ng/mL) was 47.5% in the PE group, 80% in the eclampsia group, and 10.5% in the control group (*p* = 0.04).	2^++^	B	8
Nandi et al., 2020 [61]	India	Cross-sectional	Department of Obstetrics and Gynecology, Bharati Medical College and Hospital	*n* = 50 (PE) *n* = 69 (NC)	PE	Vitamin D deficiency increases oxidative stress through alterations in one-carbon metabolism, which can result in an imbalance in LCPUFA metabolites and contribute to placental inflammation and endothelial dysfunction in PE.	2^+^	C	8
Schoenmakers et al., 2020 [62]	Sweden, 2013–2014	Nested case-control; retrospective	Antenatal care units and medical records	*n* = 1827 (cohort) *n* = 30 (normocalcemic)	Hypercalcemia crisis	Hypercalcemic women had a relatively high serum 1,25(OH)2D concentration despite appropriately suppressed PTH, which is suggestive of abnormal gestational adaptations. The prevalence of gestational hypercalcemia was 1.7% in the third trimester. Primary hyperparathyroidism and vitamin D toxicity were not found as main causes of hypercalcemia.	2^+^	C	8
Olmos-Ortiz et al., 2021 [63]	Mexico	Cross-sectional	Department of Reproductive Biology, Instituto Nacional de Ciencias Médicas y Nutrición Salvador Zubirán	*n* = 48 (UTI) *n* = 44 (normal pregnancy)	UTIs and GH	Vitamin D deficiency might predispose women to maternal cardiovascular risk and perinatal infections, especially in male-carrying pregnancies, probably owing to lower placental CYP27B1 and cathelicidin expression. Strong negative correlations were found between calcitriol and maternal systolic and diastolic blood pressure in the UTI cohort (*p* < 0.002). Cathelicidin gene expression was positively correlated with gestational age in the UTI cohort and with newborn anthropometric parameters.	2^+^	C	8

Abbreviations: 25(OH)D, 25-hydroxyvitamin D; 95% CI, 95% confidence interval; AL, allostatic load; ANOVA, analysis of variance; BMI, body mass index; CM, explant conditioned media; FDR, Food & Drug Administration; GDM, gestational diabetes mellitus; GH, gestational hypertension; HP, healthy pregnant; HUVEC, human umbilical vein endothelial cells; IRB, institutional review board; IUGR, intrauterine growth retardation; MUFA, monounsaturated fatty acids; NC, normotensive control; NOS, Newcastle–Ottawa Scale; OR, odds ratio; PE, preeclampsia or preeclamptic; PTH, parathyroid hormone; PUFA, polyunsaturated fatty acids; RFLP, restriction fragment length polymorphism; SFA, saturated fatty acids; UTI, urinary tract infection; UV, ultraviolet; VDR, vitamin D receptor; VEGF, vascular endothelial growth factor.

**Table 2:** Vitamin D-related information in original studies that show vitamin D-related risk factors for maternal morbidity during pregnancy.

Author	Factor	Vitamin D Analysis Time	Assay Method	Cutoff Values, nmol/L in Blood Sample	25(OH)D Measured or Vitamin D Supplementation Studied	Maternal Age
Rezende et al., 2012 [61]	VDR polymorphisms with PE or GH	—	Genotypes for FokI, ApaI, and BsmI determined by RFLP	—	Serum sample	27–28
Lechtermann et al., 2014 [59]	Season on maternal vitamin D status and placental vitamin D metabolism	—	ELISA; 25(OH)D ELISA (Immunodiagnostik, Bensheim, Germany)	50	Serum sample	31–32
Achkar et al., 2015 [60]	PE and vitamin D status	20 weeks	Automated chemiluminescence immunoassay (DiaSorin Liaison, Stillwater, MN, USA)	75	Serum sample	25–>35
Lawal et al., 2016 [58]	Vitamin D status and GDM	—	Cobas e411 (Roche Diagnostics, GmbH) analyzer	75	Serum sample	31.73
Mirzakhani et al., 2016 [55]	PE and vitamin D supplementation	Initiated between 10–18 weeks	Supplementation vitamin D study (4400 vs. 400 IU/day)	75	Supplementation comparison	18–39
Brodowski et al., 2017 [57]	Vitamin D status and its relationship with postpartum AL	Either 6 or 12 months postpartum	Highly selective liquid chromatography–tandem mass spectrometry using Zrt laboratory methods	50	Serum sample	27.8
Accortt et al., 2017 [56]	PE and 1,25(OH)_2_ vitamin D_3_	Delivery	LIAISON 25(OH) Vitamin D_3_ TOTAL Assay (DiaSorin, USA)	50	Maternal and cord serum sample	32.2
Singla et al., 2019 [52]	PE	—	Immune fluorescence assay test using a vitamin D kit on a Tosho AIA 360 fully automatic hormone analyzer	50	Serum sample	20–40
Nandi et al., 2020 [51]	Maternal and cord serum vitamin D levels in women with PE	Delivery	EIA method using an AC-57SF1, 25-Hydroxy Vitamin D EIA kit (AC-57SF1, IDS, Boldon, UK)	75	Maternal and cord serum sample	18–35
Rohr Thomsen et al., 2020 [54]	hypertensive disorders and PE	—	No direct measurements	—	Serum sample	<20–>35
Osman et al., 2020 [62]	Hypertensive disorders of pregnancy	—	25(OH)D_3_/D_2_ Orgentec Diagnostika ELISA Kit GmbH	50	Serum sample	20–35
Nandi et al., 2020 [53]	Maternal and cord serum vitamin D levels in women with PE	Delivery	ELISA Serum TXB2 levels (Cayman Chemicals, item No. 501020; Ann Arbor, MI, USA)	—	Maternal and cord serum sample	18–35
Schoenmakers et al., 2020 [63]	Gestational hypercalcemia	Pregnant women in trimester 1 (before gestational week 16) and in trimester 3 (after gestational week 31).	ELISA Free vitamin D (DIASource Immunoassays, Louvain-la Neuve, Belgium)	30–50	Serum sample	33.2
Olmos-Ortiz et al., 2021 [64]	Vitamin D_3_ (calcitriol active metabolite) involved in UTI	Delivery	Quantitative chemiluminescent immunoassay in the LIAISON platform	50	Serum sample	—

Abbreviations: EIA, enzyme immunoassay; ELISA, enzyme-linked immunosorbent assay; IUGR, intrauterine growth retardation; GDM, gestational diabetes mellitus; GH, gestational hypertension; PE, preeclampsia; RFLP, restriction fragment length polymorphism; UTI, urinary tract infection; VDR, vitamin D receptor.

## References

[1] Weinert L.S., Reichelt A.J., Schmitt L.R., Boff R., Oppermann M.L., Camargo J.L., Silveiro S.P. (2016). Vitamin D Deficiency Increases the Risk of Adverse Neonatal Outcomes in Gestational Diabetes. PLoS ONE.

[2] Holick M.F. (2017). The vitamin D deficiency pandemic: Approaches for diagnosis, treatment and prevention. Rev. Endocr. Metab. Disord..

[3] Feleke Y., Abdulkadir J., Mshana R., Mekbib T.A., Brunvand L., Berg J.P., Falch J.A. (1999). Low levels of serum calcidiol in an African population compared to a North European population. Eur. J. Endocrinol. Eur. Fed. Endocr. Soc..

[4] Farrant H.J., Krishnaveni G.V., Hill J.C., Boucher B.J., Fisher D.J., Noonan K., Osmond C., Veena S.R., Fall C.H. (2009). Vitamin D insufficiency is common in Indian mothers but is not associated with gestational diabetes or variation in newborn size. Eur. J. Clin. Nutr..

[5] Marcenes W., Kassebaum N.J., Bernabé E., Flaxman A., Naghavi M., Lopez A., Murray C.J. (2013). Global Burden of Oral Conditions in 1990–2010: A Systematic Analysis. J. Dent. Res..

[6] Hill K., Thomas K., AbouZahr C., Walker N., Say L., Inoue M., Suzuki E., Maternal Mortality Working Group (2007). Estimates of Maternal Mortality Worldwide between 1990 and 2005: An Assessment of Available Data. Lancet.

[7] Kilpatrick S.J. (2015). Next Steps to Reduce Maternal Morbidity and Mortality in the USA. Women’s Health.

[8] Callaghan W.M., Creanga A.A., Kuklina E.V. (2012). Severe Maternal Morbidity among Delivery and Postpartum Hospitalizations in the United States. Obstet. Gynecol..

[9] Hirshberg A., Srinivas S.K. (2017). Epidemiology of Maternal Morbidity and Mortality. Semin Perinatol..

[10] Barrera D., Díaz L., Noyola-Martínez N., Halhali A. (2015). Vitamin D and Inflammatory Cytokines in Healthy and Preeclamptic Pregnancies. Nutrients.

[11] Ross A.C., Manson J.E., Abrams S.A., Aloia J.F., Brannon P.M., Clinton S.K., Durazo-Arvizu R.A., Gallagher J.C., Gallo R.L., Jones G. (2011). The 2011 Report on Dietary Reference Intakes for Calcium and Vitamin D from the Institute of Medicine: What Clinicians Need to Know. J. Clin. Endocr..

[12] Sempos C.T., Vesper H.W., Phinney K.W., Thienpont L.M., Coates P.M., Vitamin D Standardization Program (VDSP) (2012). Vitamin D Status as an International Issue: National Surveys and the Problem of Standardization. Scand. J. Clin. Lab. Investig..

[13] Varsavsky M., Moreno P.R., Fernández A.B., Fernández I.L., Gómez J.M.Q., Rubio V.Á., Martín A.G., Berdonces M.C., Cortés S.N., Muñoz M.R. (2017). Recommended Vitamin D Levels in the General Population. Endocrinol. Diabetes Nutr..

[14] Wang W., Li G., He X., Gao J., Wang R., Wang Y., Zhao W. (2015). Serum 25-Hydroxyvitamin D Levels and Prognosis in Hematological Malignancies: A Systematic Review and Meta-Analysis. Cell. Physiol. Biochem..

[15] Cashman K.D., Dowling K.G., Škrabáková Z., Gonzalez-Gross M., Valtueña J., De Henauw S., Moreno L., Damsgaard C.T., Michaelsen K.F., Mølgaard C. (2016). Vitamin D Deficiency in Europe: Pandemic?. Am. J. Clin. Nutr..

[16] Grzegorzewska A.E., Izdebska A., Niepolski L., Warchol W., Jagodzinski P.P. (2016). Self-Reported Physical Activity, Quality of Life, and Psychological Status in Relation to Plasma 25-Hydroxyvitamin D Concentration in Patients Treated with Hemodialysis. Kidney Blood Press. Res..

[17] Yuan Y., Liu H., Ji C., Guo X., Hu L., Wen J., Cai M. (2017). Association of Maternal Serum 25-Hydroxyvitamin D Concentrations in Second Trimester with Delivery Mode in A Chinese Population. Int. J. Med. Sci..

[18] Dawson-Hughes B., Heaney R.P., Holick M.F., Lips P., Meunier P.J., Vieth R. (2005). Estimates of Optimal Vitamin D Status. Osteoporos. Int..

[19] Rosen C.J., Adams J.S., Bikle D.D., Black D.M., Demay M.B., Manson J.E., Murad M.H., Kovacs C.S. (2012). The Nonskeletal Effects of Vitamin D: An Endocrine Society Scientific Statement. Endocr. Rev..

[20] Vieth R. (2011). Why the Minimum Desirable Serum 25-Hydroxyvitamin D Level should be 75 Nmol/L (30 Ng/mL). Best Pract. Res. Clin. Endocrinol. Metab..

[21] Bischoff-Ferrari H.A., Giovannucci E., Willett W.C., Dietrich T., Dawson-Hughes B. (2006). Estimation of Optimal Serum Concentrations of 25-Hydroxyvitamin D for Multiple Health Outcomes. Am. J. Clin. Nutr..

[22] Gertner J.M., Glassman M.S., Coustan D.R., Goodman D.B. (1980). Fetomaternal Vitamin D Relationships at Term. J. Pediatr..

[23] Delvin E.E., Glorieux F.H., Salle B.L., David L., Varenne J.P. (1982). Control of Vitamin D Metabolism in Preterm Infants: Feto-Maternal Relationships. Arch. Dis. Child..

[24] O’Brien K.O., Li S., Cao C., Kent T., Young B.V., Queenan R.A., Pressman E.K., Cooper E.M. (2014). Placental CYP27B1 and CYP24A1 Expression in Human Placental Tissue and their Association with Maternal and Neonatal Calcitropic Hormones. J. Clin. Endocrinol. Metab..

[25] Hollis B.W., Pittard W.B. (1984). Evaluation of the Total Fetomaternal Vitamin D Relationships at Term: Evidence for Racial Differences. J. Clin. Endocrinol. Metab..

[26] Young B.E., McNanley T.J., Cooper E.M., McIntyre A.W., Witter F., Harris Z.L., O’Brien K.O. (2012). Vitamin D Insufficiency is Prevalent and Vitamin D is Inversely Associated with Parathyroid Hormone and Calcitriol in Pregnant Adolescents. J. Bone Miner. Res..

[27] Institute of Medicine (IOM) (2011). Dietary Reference Intakes for Calcium and Vitamin D.

[28] Heaney R.P., Holick M.F. (2011). Why the IOM recommendations for vitamin D are deficient. J. Bone Miner. Res..

[29] Holick M.F. (2011). Vitamin D: A D-Lightful Solution for Health. J. Investig. Med..

[30] Singh S., Garg R., Meena A., Kumar D. (2021). Perinatal Outcome in Vitamin D Deficiency and Effect of Oral and Intramuscular Vitamin D3 Supplementation in Antenatal Women on Pregnancy Outcomes. J. South Asian Feder. Obst. Gyne..

[31] Hollis B.W., Wagner C.L., Howard C.R., Ebeling M., Shary J.R., Smith P.G., Taylor S.N., Morella K., Lawrence R.A., Hulsey T.C. (2015). Maternal Versus Infant Vitamin D Supplementation During Lactation: A Randomized Controlled Trial. Pediatrics.

[32] McDonnell S.L., Baggerly K.A., Baggerly C.A., Aliano J.L., French C.B., Baggerly L.L., Ebeling M.D., Rittenberg C.S., Goodier C.G., Niño J.F. (2017). Maternal 25(OH)D concentrations ≥40 ng/mL associated with 60% lower preterm birth risk among general obstetrical patients at an urban medical center. PLoS ONE.

[33] Olmos-Ortiz A., Avila E., Durand-Carbajal M., Díaz L. (2015). Regulation of Calcitriol Biosynthesis and Activity: Focus on Gestational Vitamin D Deficiency and Adverse Pregnancy Outcomes. Nutrients.

[34] Tamblyn J., Hewison M., Wagner C., Bulmer J., Kilby M. (2015). Immunological Role of Vitamin D at the Maternal–fetal Interface. J. Endocrinol..

[35] Ma R., Gu Y., Zhao S., Sun J., Groome L.J., Wang Y. (2012). Expressions of Vitamin D Metabolic Components VDBP, CYP2R1, CYP27B1, CYP24A1, and VDR in Placentas from Normal and Preeclamptic Pregnancies. Am. J. Physiol. Endocrinol. Metab..

[36] Díaz L., Arranz C., Avila E., Halhali A., Vilchis F., Larrea F. (2002). Expression and Activity of 25-Hydroxyvitamin D-1α-Hydroxylase are Restricted in Cultures of Human Syncytiotrophoblast Cells from Preeclamptic Pregnancies. J. Clin. Endocrinol. Metab..

[37] Pospechova K., Rozehnal V., Stejskalova L., Vrzal R., Pospisilova N., Jamborova G., May K., Siegmund W., Dvorak Z., Nachtigal P. (2009). Expression and Activity of Vitamin D Receptor in the Human Placenta and in Choriocarcinoma BeWo and JEG-3 Cell Lines. Mol. Cell. Endocrinol..

[38] Slominski A.T., Kim T., Shehabi H.Z., Semak I., Tang E.K., Nguyen M.N., Benson H.A., Korik E., Janjetovic Z., Chen J. (2012). In Vivo Evidence for a Novel Pathway of Vitamin D3 Metabolism Initiated by P450scc and Modified by CYP27B1. FASEB J..

[39] Aguilar-Cordero M., Lasserrot-Cuadrado A., Mur-Villar N., León-Ríos X., Rivero-Blanco T., Pérez-Castillo I. (2020). Vitamin D, Preeclampsia and Prematurity: A Systematic Review and Meta-Analysis of Observational and Interventional Studies. Midwifery.

[40] Marino R., Misra M. (2019). Extra-Skeletal Effects of Vitamin D. Nutrients.

[41] Larque E., Morales E., Leis R., Blanco-Carnero J.E. (2018). Maternal and Foetal Health Implications of Vitamin D Status during Pregnancy. Ann. Nutr. Metab..

[42] Wei S., Qi H., Luo Z., Fraser W.D. (2013). Maternal Vitamin D Status and Adverse Pregnancy Outcomes: A Systematic Review and Meta-Analysis. J. Matern. Fetal. Neonatal. Med..

[43] Dawodu A., Akinbi H. (2013). Vitamin D Nutrition in Pregnancy: Current Opinion. Int. J. Woman’s Health.

[44] Schöttker B., Ball D., Gellert C., Brenner H. (2013). Serum 25-Hydroxyvitamin D Levels and overall Mortality. A Systematic Review and Meta-Analysis of Prospective Cohort Studies. Ageing Res. Rev..

[45] Moher D., Shamseer L., Clarke M., Ghersi D., Liberati A., Petticrew M., Shekelle P., Stewart L.A. (2015). Preferred Reporting Items for Systematic Review and Meta-Analysis Protocols (PRISMA-P) 2015 Statement. Syst. Rev..

[46] Moher D., Liberati A., Tetzlaff J., Altman D.G. (2009). Preferred Reporting Items for Systematic Reviews and Meta-Analyses: The PRISMA Statement. Ann. Intern. Med..

[47] Wells G.A., Tugwell P., O’Connell D., Welch V., Peterson J., Shea B., Losos M. The Newcastle-Ottawa Scale (NOS) for Assessing the Quality of Nonrandomized Studies in Meta-Analyses. Syst. Rev..

[48] Stang A. (2010). Critical Evaluation of the Newcastle-Ottawa Scale for the Assessment of the Quality of Nonrandomized Studies in Meta-Analyses. Eur. J. Epidemiol..

[49] Scottish Intercollegiate Guidelines Network (SIGN) (2015). SIGN 50: A Guideline Developer’s Handbook.

[50] Sackett D.L. (1997). Evidence-Based Medicine. Semin. Perinatol..

[51] Nandi A., Wadhwani N., Randhir K., Wagh G., Joshi S. (2020). Association of Vitamin D with Fatty Acids in Pregnancy. Prostaglandins Leukot. Essent. Fat. Acids.

[52] Jeyakumar A., Shinde V., Ravindran R. (2021). Pooled estimate of Vitamin D deficiency among pregnant women in India: A systematic review and meta-analysis. J. Heath Popul. Nutr..

[53] Nandi A.A., Wadhwani N.S., Randhir K.N., Madiwale S.D., Deshpande J.S., Wagh G.N., Joshi S.R. (2021). Maternal vitamin D deficiency influences long-chain polyunsaturated fatty acids and pregnancy outcome in association with alterations in one-carbon metabolism. Nutr. Res..

[54] Rohr Thomsen C., Brink Henriksen T., Uldbjerg N., Milidou I. (2020). Seasonal Variation in the Hypertensive Disorders of Pregnancy in Denmark. Acta Obstet. Gynecol. Scand..

[55] Mirzakhani H., Litonjua A.A., McElrath T.F., O’Connor G., Lee-Parritz A., Iverson R., Macones G., Strunk R.C., Bacharier L.B., Zeiger R. (2016). Early Pregnancy Vitamin D Status and Risk of Preeclampsia. J. Clin. Investig..

[56] Accortt E.E., Mirocha J., Schetter C.D., Hobel C.J. (2017). Adverse Perinatal Outcomes and Postpartum Multi-Systemic Dysregulation: Adding Vitamin D Deficiency to the Allostatic Load Index. Matern. Child Health J..

[57] Brodowski L., Burlakov J., Hass S., von Kaisenberg C., von Versen-Höynck F. (2017). Impaired Functional Capacity of Fetal Endothelial Cells in Preeclampsia. PLoS ONE.

[58] Lawal O.A. (2016). A Study of Vitamin D Status of Women with Gestational Diabetes Mellitus in Abuja, Nigeria. Fac. Pathol..

[59] Lechtermann C., Hauffa B.P., Herrmann R., Schündeln M.M., Gellhaus A., Schmidt M., Grasemann C. (2014). Maternal Vitamin D Status in Preeclampsia: Seasonal Changes are Not Influenced by Placental Gene Expression of Vitamin D Metabolizing Enzymes. PLoS ONE.

[60] Achkar M., Dodds L., Giguère Y., Forest J., Armson B.A., Woolcott C., Agellon S., Spencer A., Weiler H.A. (2015). Vitamin D Status in Early Pregnancy and Risk of Preeclampsia. Obstet. Gynecol..

[61] Rezende V.B., Sandrim V.C., Palei A.C., Machado L., Cavalli R.C., Duarte G., Tanus-Santos J.E. (2012). Vitamin D Receptor Polymorphisms in Hypertensive Disorders of Pregnancy. Mol. Biol. Rep..

[62] Osman O.M., Gaafar T., Eissa T.S., Abdella R., Ebrashy A., Ellithy A. (2020). Prevalence of Vitamin D Deficiency in Egyptian Patients with Pregnancy-Induced Hypertension. J. Perinat. Med..

[63] Schoenmakers I., Piec I., Baban S., Barebring L., Green D., Washbourne C.J., Tang J.C.Y., Fraser W.D., Augustin H. (2020). Gestational hypercalcemia: Prevalence and biochemical profile. J. Steroid Biochem. Mol. Biol..

[64] Olmos-Ortiz A., Olivares-Huerta A., García-Quiroz J., Avila E., Halhali A., Quesada-Reyna B., Larrea F., Zaga-Clavellina V., Díaz L. (2021). Cord serum calcitriol inversely correlates with maternal blood pressure in urinary tract infection-affected pregnancies: Sex-dependent immune implications. Nutrients.

[65] de Souza E.A., Pisani L.P. (2020). The Relationship among Vitamin D, TLR4 Pathway and Preeclampsia. Mol. Biol. Rep..

[66] Nandi A.A., Wadhwani N.S., Joshi S.R. (2017). Altered Metabolic Homeostasis between Vitamin D and Long Chain Polyunsaturated Fatty Acids in Preeclampsia. Med. Hypotheses.

[67] Agarwal S., Kovilam O., Agrawal D.K. (2018). Vitamin D and its Impact on Maternal-Fetal Outcomes in Pregnancy: A Critical Review. Crit. Rev. Food Sci. Nutr..

[68] Palacios C., De-Regil L.M., Lombardo L.K., Peña-Rosas J.P. (2016). Vitamin D Supplementation during Pregnancy: Updated Meta-Analysis on Maternal Outcomes. J. Steroid Biochem. Mol. Biol..

[69] Pérez-López F.R., Pasupuleti V., Mezones-Holguin E., Benites-Zapata V.A., Thota P., Deshpande A., Hernandez A.V. (2015). Effect of Vitamin D Supplementation during Pregnancy on Maternal and Neonatal Outcomes: A Systematic Review and Meta-Analysis of Randomized Controlled Trials. Fertil. Steril..

[70] Weinert L.S., Silveiro S.P. (2015). Maternal–fetal Impact of Vitamin D Deficiency: A Critical Review. Matern. Child Health J..

[71] Girling J., Sykes L. (2013). Thyroid Disorders and Other Endocrinological Disorders in Pregnancy. Obstet. Gynaecol. Reprod. Med..

[72] Tabesh M., Salehi-Abargouei A., Tabesh M., Esmaillzadeh A. (2013). Maternal Vitamin D Status and Risk of Pre-Eclampsia: A Systematic Review and Meta-Analysis. J. Clin. Endocrinol. Metab..

[73] Alzaim M., Wood R.J. (2013). Vitamin D and Gestational Diabetes Mellitus. Nutr. Rev..

[74] Senti J., Thiele D.K., Anderson C.M. (2012). Maternal Vitamin D Status as a Critical Determinant in Gestational Diabetes. J. Obstet. Gynecol. Neonatal. Nurs..

[75] Barrett H., McElduff A. (2010). Vitamin D and Pregnancy: An Old Problem Revisited. Best Pract. Res. Clin. Endocrinol. Metabo..

[76] Mulligan M.L., Felton S.K., Riek A.E., Bernal-Mizrachi C. (2010). Implications of Vitamin D Deficiency in Pregnancy and Lactation. Obstet. Gynecol..

[77] Thorne-Lyman A., Fawzi W.W. (2012). Vitamin D during Pregnancy and Maternal, Neonatal and Infant Health Outcomes: A Systematic Review and Meta-Analysis. Paediatr. Perinat. Epidemiol..

[78] Baker A.M., Haeri S., Camargo C.A., Espinola J.A., Stuebe A.M. (2010). A nested case-control study of midgestation vitamin D deficiency and risk of se-vere preeclampsia. J. Clin. Endocrinol. Metab..

[79] Powe C.E., Seely E.W., Rana S., Bhan I., Ecker J., Karumanchi S.A., Thadhani R. (2010). First trimester vitamin D, vitamin D binding protein, and subsequent preeclampsia. Hypertension.

[80] Shand A., Nassar N., Von Dadelszen P., Innis S., Green T. (2010). Maternal vitamin D status in pregnancy and adverse pregnancy outcomes in a group at high risk for pre-eclampsia. BJOG Int. J. Obstet. Gynaecol..

[81] Holick M.F., Chen T.C. (2008). Vitamin D Deficiency: A Worldwide Problem with Health Consequences. Am. J. Clin. Nutr..

[82] Holick M.F. (2007). Vitamin D Deficiency. N. Engl. J. Med..

[83] Baggerly C.A., Cuomo R.E., French C.B., Garland C.F., Gorham E.D., Grant W.B., Heaney R.P., Holick M.F., Hollis B.W., McDonnell S.L. (2015). Sunlight and Vitamin D: Necessary for Public Health. J. Am. Coll. Nutr..

[84] Bodnar L.M., Catov J.M., Simhan H.N., Holick M.F., Powers R.W., Roberts J.M. (2007). Maternal Vitamin D Deficiency Increases the Risk of Preeclampsia. J. Clin. Endocrinol. Metab..

[85] Bui T., Christin-Maitre S. (2011). Vitamin D and Pregnancy. Ann. Endocrinol..

[86] Brannon P.M. (2012). Vitamin D and Adverse Pregnancy Outcomes: Beyond Bone Health and Growth. Proc. Nutr. Soc..

[87] Palaniswamy S., Williams D., Järvelin M., Sebert S. (2015). Vitamin D and the Promotion of Long-Term Metabolic Health from a Programming Perspective. Nutr. Metab. Insights.

[88] Saraf R., Morton S.M., Camargo C.A., Grant C.C. (2016). Global Summary of Maternal and Newborn Vitamin D Status—A Systematic Review. Matern. Child Nutr..

[89] De-Regil L.M., Palacios C., Lombardo L.K., Peña-Rosas J.P. (2016). Vitamin D Supplementation for Women during Pregnancy. Cochrane Database Syst. Rev..

[90] Hollis B.W., Johnson D., Hulsey T.C., Ebeling M., Wagner C.L. (2011). Vitamin D Supplementation during Pregnancy: Double-blind, Randomized Clinical Trial of Safety and Effectiveness. J. Bone Miner. Res..

[91] World Health Organization (WHO) (2021). Vitamin D Supplementation during Pregnancy.

[92] American Pregnancy Association (2021). Vitamin D and Pregnancy.

[93] Hong-Bi S., Yin X., Xiaowu Y., Ying W., Yang X., Ting C., Na W. (2018). High Prevalence of Vitamin D Deficiency in Pregnant Women and its Relationship with Adverse Pregnancy Outcomes in Guizhou, China. J. Int. Med. Res..

[94] Hynes C., Jesurasa A., Evans P., Mitchell C. (2017). Vitamin D Supplementation for Women before and during Pregnancy: An Update of the Guidelines, Evidence, and Role of GPs and Practice Nurses. Br. J. Gen. Pract..

[95] Lötscher Q., l’Alemand D., Bischoff-Ferrari H., Burckhardt P. (2012). Vitamin D Deficiency: Evidence, Safety, and Recommendations for the Swiss Population. https://www.zora.uzh.ch/id/eprint/73029/.

[96] Health Canada (2021). Vitamin D and Calcium: Updated Dietary Reference Intakes. https://www.canada.ca/en/health-canada/services/food-nutrition/healthy-eating/vitamins-minerals/vitamin-calcium-updated-dietary-reference-intakes-nutrition.html.

[97] Özdemir A.A., Gündemir Y.E., Küçük M., Sarıcı D.Y., Elgörmüş Y., Çağ Y., Bilek G. (2018). Vitamin D Deficiency in Pregnant Women and their Infants. J. Clin. Pediatr. Endocrinol..

[98] Keshavarz P., Jandaghi P., Shafiee M., Islam N., Vatanparast H. (2020). Maternal Vitamin D Status among Different Ethnic Groups and its Potential Contribution to Adverse Pregnancy and Child Outcomes. Vitamin D Deficiency.

[99] Ministry of Health (2021). Companion Statement on Vitamin D and Sun Exposure in Pregnancy and Infancy in New Zealand: A Supplement to the Consensus Statement on Vitamin D and Sun Exposure in New Zealand. https://www.health.govt.nz/publication/companion-statement-vitamin-d-and-sun-exposure-pregnancy-and-infancy-new-zealand.

